# Phase I/II storm study: Intravenous delivery of a novel oncolytic immunotherapy agent, Coxsackievirus A21, in advanced cancer patients

**DOI:** 10.1186/2051-1426-3-S2-P341

**Published:** 2015-11-04

**Authors:** Hardev Pandha, Kevin Harrington, Christy Ralph, Alan Melcher, Mark Grose, Darren Shafren

**Affiliations:** 1 University of Surrey, Guildford, UK; 2 Institute of Cancer Research and Royal Marsden Hospital, London, UK; 3 St. James's Institute of Oncology, St. James's University Hospital, Leeds, UK; 4 Viralytics Limited, Toronto, ON, Canada; 5 Viralytics, Sydney, Australia

## Background

Coxsackievirus A21 (CVA21) is a naturally occurring “common cold” intercellular adhesion molecule-1 (ICAM-1)-targeted RNA virus. Surface ICAM-1 is up-regulated on a number of cancers including melanoma, non-small cell lung, bladder and prostate cancers. CAVATAK is a novel bio-selected formulation of CVA21, which displays potent oncolytic activity against *in vitro* cultures of cancer cells and *in vivo* xenografts of a number of cancers. In this Phase I/II study advanced cancer patients received multiple intravenous (IV) doses of CAVATAK to assess treatment tolerance, levels of viral replication and viral-induced immune activation within the tumor micro-environment.

## Methods

The Phase I/II STORM (Systemic Treatment Of Resistant Malignancies: NCT02043665) study is investigating the tolerance of multiple escalating IV doses of CVA21 in approximately 30 advanced cancer patients. In cohort 1 (n=3), patients were infused with CVA21 at a dose of 1 × 10^8^ TCID_50_, in cohort 2 (n=3), patients were infused with CVA21 at a dose of 3 × 10^8^ TCID_50_ and treatment of patients in Cohort 3 (n=12-18) with CVA21 at a dose of 1 × 10^9^ TCID_50_ has commenced. Tumor biopsies at 8 days following the initial CVA21 infusion are being monitored for levels of virus and markers of potential immune activation. Sequential serum samples are being analyzed for viral loads, kinetics of anti-CVA21 neutralizing antibody (nAb) development and immune system activation via relative serum levels of a panel of immune cytokines/cell subsets.

## Results

To date multi-dose intravenous administration to patients in Cohorts 1,2 and 3 has been well tolerated, with no Grade 3 or 4 product-related AE's. Preliminary data indicate that the prolonged presence of serum CVA21 RNA in some, but not all, patients at times (up to 4 days post-infusion), when complete decay of the administered viral dose was expected may indicate possible viral replication within the tumor . Evidence of CVA21 tumor targeting is confirmed with 2 of 2 melanoma patients in Cohort 3 displaying replicating CVA21 in tumor biopsies. Such replication in pre-clinical melanoma xenograft models was potentially immunogenic, as evidenced by gene expression increases of CXCL-10 and PD-L1. The interim dataalso highlight a robust “multi-dosing-window” in the absence of significant levels of nAb for approximately 7 days post initial viral infusion.

## Conclusions

Preliminary data offer an exciting possibly that tumor targeting, infection and immune activation mediated by IV CVA21 may lead to increases in anti-tumor activity, particularly when in future used in combination with immune checkpoint blockade.

